# Whole genome sequencing reveals potential targets for therapy in patients with refractory *KRAS* mutated metastatic colorectal cancer

**DOI:** 10.1186/1755-8794-7-36

**Published:** 2014-06-18

**Authors:** Vijayalakshmi Shanmugam, Ramesh K Ramanathan, Nicole A Lavender, Shripad Sinari, Manpreet Chadha, Winnie S Liang, Ahmet Kurdoglu, Tyler Izatt, Alexis Christoforides, Hollie Benson, Lori Phillips, Angela Baker, Christopher Murray, Galen Hostetter, Daniel D Von Hoff, David W Craig, John D Carpten

**Affiliations:** 1 Translational Genomics Research Institute (TGen), 445 N Fifth Street, Phoenix, AZ 85004, USA; 2 Virginia G Piper Cancer Center, Scottsdale, AZ, USA; 3 Van Andel Research Institute (VARI), Grand Rapids, MI, USA

**Keywords:** Metastatic colorectal cancer, Whole genome sequencing, *KRAS* mutations

## Abstract

**Background:**

The outcome of patients with metastatic colorectal carcinoma (mCRC) following first line therapy is poor, with median survival of less than one year. The purpose of this study was to identify candidate therapeutically targetable somatic events in mCRC patient samples by whole genome sequencing (WGS), so as to obtain targeted treatment strategies for individual patients.

**Methods:**

Four patients were recruited, all of whom had received > 2 prior therapy regimens. Percutaneous needle biopsies of metastases were performed with whole blood collection for the extraction of constitutional DNA. One tumor was not included in this study as the quality of tumor tissue was not sufficient for further analysis. WGS was performed using Illumina paired end chemistry on HiSeq2000 sequencing systems, which yielded coverage of greater than 30X for all samples. NGS data were processed and analyzed to detect somatic genomic alterations including point mutations, indels, copy number alterations, translocations and rearrangements.

**Results:**

All 3 tumor samples had *KRAS* mutations, while 2 tumors contained mutations in the *APC* gene and the *PIK3CA* gene. Although we did not identify a *TCF7L2-VTI1A* translocation, we did detect a *TCF7L2* mutation in one tumor. Among the other interesting mutated genes was *INPPL1,* an important gene involved in PI3 kinase signaling. Functional studies demonstrated that inhibition of *INPPL1* reduced growth of CRC cells, suggesting that *INPPL1* may promote growth in CRC.

**Conclusions:**

Our study further supports potential molecularly defined therapeutic contexts that might provide insights into treatment strategies for refractory mCRC. New insights into the role of *INPPL1* in colon tumor cell growth have also been identified. Continued development of appropriate targeted agents towards specific events may be warranted to help improve outcomes in CRC.

## Background

Colorectal cancer (CRC) is one of the most common cancers in the United States with an estimated 150,000 new cases and 50,000 deaths each year [1]. While early stage CRC (stage I and II) has a high cure rate after surgery, the recurrence rate is about 50% for stage III CRC after surgery alone and most patients with metastatic disease will ultimately succumb to their cancer [2]. Chemotherapy is the primary treatment for metastatic disease. Currently, there are roughly 5 classes of approved drugs for treating mCRC [3]. These agents include: (1) Fluoropyrimidines: 5-FU and capecitabine (2) Platinum derivative: oxaliplatin (3) Camptothecin derivative: irinotecan (4) EGFR inhibitors: cetuximab and panitumumab and (5) VEGF inhibitors: bevacizumab, afibercept and regorafenib. *EGFR* inhibitors represent “targeted agents” and their use is limited to about 60% of tumors, which have wild type *KRAS* genotype. These agents are given in combination, and ultimately patients with *KRAS* mutations run out of treatment options after 2–3 lines of therapy, with a commonly used sequence being a combination of 5-FU, oxaliplatin and bevacizumab (FOLFOX + bevacizumab) followed by 5-FU/irinotecan (FOLFIRI) with the addition of bevacizumab or afibercept, and the recently approved agent, regorafenib as a third line option in some patients [4].

A number of molecular targets and pathways have been described in CRC. Aberrations in chromosome instability and mismatch repair have been widely identified in a number of cases (85%) [5] with mutations in *APC* and MutL-homolog (MLH) genes. Mutations in *TP53* have been found in about 50% of colorectal cancers globally [6] as p53 plays key roles in cell regulation, apoptosis, DNA repair and differentiation. *KRAS* mutations are also common in CRC, and occur at a frequency of ~ 40% [7]. Several other pathways which also trigger the malignant phenotype include the *TGFβ* signaling pathway mediated through downstream targets such as *SMAD2* and *SMAD4*, and components of *RAS/MAPK*, *JNK* and *PI3K*/*AKT* pathways [8]. Studies of protein coding genomic sequences of colorectal cancers revealed that only a subset of these genes actually contribute to the process of carcinogenesis whereas a vast majority of them actually affect other cellular processes such as transcription, adhesion and invasion [9]. Sequencing studies of the mutational landscape of colorectal cancer revealed that the mutational spectrum is comprised of a limited number of frequently mutated genes and a large number of infrequently mutated genes [10]. Furthermore, a previous sequencing study of 9 colorectal cancers and matched normal tissues reported additional recurrent events, notably a *VTI1A-TCF7L2* fusion gene present in ~ 3% of the patients [11].

Personalized CRC patient treatment based on characterizing the individual tumors by high throughput sequencing strategies has been attempted [12]. Most studies reporting sequencing data have been with panels of select genes, or with cell lines, patient derived xenografts, or primary tumors removed at surgery [13]. Our group has instituted a pilot program to sequence various solid tumors in patients with refractory solid tumors [14,15]. In obtaining clinically relevant information that can be of use by the treating physician, tumor biopsies are obtained in patients with advanced solid tumors refractory to approved therapies [16,17]. In our current study, we utilized next generation sequencing technologies (NGS) to identify potential biomarkers so as to identify treatment options for patients with mCRC.

## Methods

### Participants & samples

All patients signed an IRB approved consent form prior to participation at the Virginia G. Piper Cancer Center, Scottsdale Healthcare, Scottsdale, AZ. Fresh frozen tumor biopsy specimens were collected and quality assessed for tumor cellularity, necrosis, crush artifact, etc. A blood sample was also provided for the collection of constitutional genomic DNA. RNA and DNA were extracted from tumor biopsy specimens using the Qiagen All Prep kit (Qiagen, Germantown, MD). Germline DNA (Table 1) provides information regarding patients and samples.

**Table 1:** Patient clinical information

	**CLN2**	**CLN3**	**CLN4**
Age at diagnosis	73	45	50
Gender	male	male	male
Ethnicity	Caucasian	Caucasian	Hispanic
Diagnosis	Colon adenocarcinoma	Rectal adenocarcinoma	Colon adenocarcinoma
Tumor cellularity	60%	50%	30%
Sequenced biopsy	Liver metastasis	Right gluteal mass	Right abdominal mass

### Genomic DNA isolation

#### Fresh frozen tissue

Tissue from the needle biopsy was disrupted and homogenized in Buffer RLT plus, Qiagen AllPrep DNA/RNA Mini Kit, using the Bullet Blender™, Next Advance. Specifically, tissue was transferred to a microcentrifuge tube containing 600 μl of Buffer RLT plus, and stainless steel beads. The tissue was homogenized in the Bullet Blender at room temperature. The sample was centrifuged at full speed and the lysate was transferred to the Qiagen AllPrep DNA spin column. Genomic DNA purification was conducted as directed by the AllPrep DNA/RNA Mini Handbook, Qiagen. DNA was quantified using the Nanodrop spectrophotometer and quality was accessed from 260/280 and 260/230 absorbance ratios.

#### Blood

The QIAamp DNA Blood Maxi Kit, Qiagen, was used to isolate DNA from 8–10 ml of whole blood. The protocol was conducted as written. Specifically, the buffy coat layer was isolated from whole blood by centrifugation. The volume of buffy coat was brought up to 5–10 ml with PBS and treated with Qiagen protease at 70°C. 100% ethanol was added and the sample was applied to a QIAamp Maxi column and centrifuged. Samples were then washed with buffers AW1 and AW2 and eluted in 1000 μl of Buffer AE. The Qubit 2.0 Fluorometer, Invitrogen, and the Nanodrop spectrophotometer, Thermo Scientific, were used to assess DNA quality and concentration.

### Sequencing data analysis

#### Illumina whole genome sequencing

DNA libraries were prepared using the NEBNext DNA Sample Prep Master Mix Set (New England Biolabs, Ipswich, MA). For each sample library preparation, 1 μg of high molecular weight genomic DNA was fragmented using the Covaris S2 system. Fragmented samples were end repaired using T4 DNA polymerase, DNA polymerase I Klenow fragment, and T4 polynucleotide kinase. Samples were next adenylated using Klenow fragment 3′-5′ exo minus enzyme, ligated with Illumina adapters, size selected at 350-450 bp, and PCR amplified using Phusion High-Fidelity PCR Master Mix w/HF buffer (New England Biolabs). The DNA libraries were clustered onto flowcells using Illumina’s cBot and HiSeq Paired End Cluster Generation kits as per manufacturer protocol (Illumina, San Diego, CA). NGS of CLN2 and CLN3 samples were carried out using the Illumina HiSeq 2000 platform using the v1.5 chemistry reagents and flowcells. The CLN4 sample was sequenced on the Illumina HiSeq 2000 platform using the v3 chemistry. The total length of each paired end sequencing run was 200 cycles.

#### Single nucleotide variant detection

Somatic single nucleotide variant calling was performed using SolSNP [18] and Mutation Walker. SolSNP, an individual sample variant detector (classifier) implemented in Java does the variant calling based on a modified Kolmogorov-Smirnov like statistic, which incorporates base quality scores. The algorithm is non-parametric and makes no assumptions on the nature of the data. It compares the discrete sampled distribution, the pileup on each strand, to the expected distributions (according to ploidy). In case of diploid genome, both strands need to provide evidence for the variation. Zero quality bases are trimmed off the pileup before the comparisons. An important aspect of SolSNP that reduces overcalling inherent to the K-S statistic algorithm is that filters are included to reduce false positive rates, of which both strands must provide evidence for the variation.

While making somatic calls, SolSNP’s high quality genotype call is made for all callable loci of the normal sample. To reduce false negatives, variant loci in tumor samples are called with the Variant Consensus mode. Variant loci in tumor samples that exhibit a high quality homozygous reference genotype in the normal sample are considered as somatic. To call somatic variants, SolSNP is augmented by a Python script.

Mutation Walker, an in house tool developed in Java, utilizes the variant discovery tools from Genome Analysis Toolkit (GATK) [19] as a framework. SNPs that were called using both tools were compiled and further examined. Two sets of thresholds, strict and lenient, were enabled to reduce the false negative rate. Data from each of these two sets were visually examined for false positives to generate a final filtered list of true SNVs, which were annotated with GENCODE using an internal annotation script.

#### Copy number analysis

For copy number analysis, a custom tool was developed based on a sliding window comparison of coverage for tumor/normal. This method has been adopted by Liang *et al.* for their analysis [16]. Copy number gains and losses were calculated from log2 difference in normalized physical coverage between germline and tumor samples across a sliding window of 2 kb, where physical coverage was incremented for the length of the insert between the read pairs for insert sizes less than 3 standard deviations of the mean. High repeat regions such as centromeres were defined as regions where the log2 normalized coverage exceeded 3 in the germline sample and were thus excluded. Regions where the coverage was zero were replaced by 1 so that homozygous deletions avoid infinite values and are generally capped at approximately −3.

#### Indel (Insertion/deletion) detection

For detecting somatic indels we employed a two-step strategy. In the first step we removed from the tumor sample bam, reads whose insert size lay outside a 50 bp - 500 bp interval from the tumor bam files. GATK [19] is then used to generate a list of potential small indels from this bam. A customized perl script, which uses the Bio-SamTools library from BioPerl [20], takes these indel positions and for each of the indels looks at the region in the normal sample consisting of 5 bp upstream from the start and 5 bp downstream from the end of the indel. An indel is determined to be somatic only if there was no indel detected in the region under consideration in the normal DNA.

#### Translocation detection

A series of customized perl scripts are employed in the detection of translocation. These scripts use SAMtools [21] internally to access the bam files. The algorithm consists of two steps. The first is to detect potential translocation in both tumor and normal samples. The second is a comparison of potential translocations in tumor to those detected in the normal sample to weed out potential false positives. The detection of a potential translocation is an exercise in outlier detection. We take a sliding window of 2kbp and count the discordant reads, whose mates align on a different chromosome. We use 2kbp, as it is close to the mean of the estimated insert size distribution, and gives the best resolution for the detection of an interchromosomal translocation. For each window we choose the highest hit to be the chromosome to which mates of most of the discordant reads mapped. For purposes of brevity, we call the subset of discordant reads whose mate maps to the highest hit in the window as the hit discordant reads. We compare the ratios of the hit discordant reads, to the total aligned reads, across all the windows to detect potential outliers. Outlier detection is performed under the assumption that the distribution, of the proportion of hit discordant reads in a 2 kb window aggregates across the chromosome, and will follow a normal distribution. We then compute the mean of this distribution and choose a cutoff of 3 standard deviations. The window with a proportion of hit discordant reads, higher than this cutoff contains the region of potential translocation. The actual region of translocation is then determined by the span of the hit discordant reads in the window. For somatic translocations, the normal and the tumor sample are called separately and regions of overlap are eliminated. These regions were further inspected visually to reduce false positives to arrive at the most confident list.

#### SIFT analysis

Single nucleotide variations identified from paired tumor normal analysis were analyzed using the SIFT algorithm. Non-synonymous SNPs in the coding region were checked to see if an amino acid substitution in the protein could actually be damaging by altering the function of the protein [22].

#### Cell culture conditions and treatments

X-MAN HCT116 cell line was purchased from Horizon Discovery Ltd. (Cambridge, UK) and cultured in McCoy’s 5A modified media with 10% FBS (Life Technologies, Grand Island, NY). HEK293 cells were obtained from the American Type Culture Collection (ATCC) and passaged in RPMI with 10% FBS and 100 units insulin (Sigma-Aldrich, St. Louis, MO). Inhibition of *INPPL1* expression was achieved through the use of siRNA sequences (Qiagen, Valencia, CA). siRNA transfections were performed with Lipofectamine 2000 (Life Technologies) and pooled *INPPL1* (FlexiTube GeneSolution GS3636) siRNA sequences [Qiagen]. Non-silencing negative control and GFP siRNA sequences were utilized as negative controls (Qiagen). AllStars Hs Cell Death and UBBs1 sequences served as positive controls to assess transfection efficiency (Qiagen). Cells were seeded in 384-well pates containing siRNA sequences (i.e., reverse transfection) and proliferation was measured via CyQUANT Direct Cell Proliferation Assay (Life Technologies). Changes in proliferation were assessed based on comparison of non-silencing siRNA controls to target siRNA. Data shown for siRNA experiments was generated using Excel version 14.3.5 (Microsoft Corporation, Redmond, WA). For siRNA data, differences in proliferation were evaluated by T Test and p values ≤ 0.05 were considered statistically significant.

#### Cell lysates and immunoblotting

Untreated as well as transfected whole cell lysates were prepared using the cOmplete Lysis-M kit (Roche Applied Science, Indianapolis, IN). Protein samples were prepared by combining lysates with NuPAGE Sample Reducing Agent, NuPAGE LDS Sample Buffer, and nuclease-free water (Life Technologies) and then boiling for 5 minutes at 100**°**C. Samples were loaded onto NuPAGE 4-12% Bis Tris Gels (Life Technologies), separated by SDS-PAGE, and then transferred to PVDF membranes. Proteins were blocked in 5% non-fat milk and incubated with the appropriate antibody in 5% BSA (Sigma-Aldrich). The following antibodies were used for Western blot analyses: AKT (#9272), pAKT Thr308 (#4056), pAKT Ser473 (#4058), CASP7 (#9492), Fox01 (#2880), pFox01 Ser256 (#9461), GSK-3β (#9315), pGSK-3β Ser9 (#9336), INNPL1 (#2839), p70 S6 Kinase (#2708), PDK1 (#5662), pPDK1 Ser241 (#3438), PI3K p85 (#4292), and GAPDH (#2118) [Cell Signaling, Danvers MA]; and pGSK-3β Tyr216 (#75745) [Abcam Inc., Cambridge, MA]. Protein bands were visualized using Pierce ECL Western Blotting Substrate (Thermo Fisher Scientific, Rockford, IL).

## Results

### Clinical history

Four patients were recruited for this study. In patient CLN1, the tumor sample was not considered adequate for NGS and results are not presented. Clinical information is provided in Table 1.

Patient CLN2 is a 73 year old Caucasian male first diagnosed with a T3N2 moderately differentiated adenocarcinoma of the ascending colon. He received adjuvant therapy with FOLFOX but had rapid recurrence of disease 5 months after initial surgery. The patient had subsequent therapy with irinotecan/bevacizumab and then gemcitabine, mitomycin and insulin potentiating therapy. At time of referral, there was metastatic disease in the pelvis and multiple omental lesions. A CT guided biopsy of a nodule in the right side of the abdomen was performed. The biopsy from this mass (60% tumor cellularity) was consistent with the colon primary.

Patient CLN3 is a 45 year old Caucasian male first diagnosed with a T2NO rectal cancer for which primary surgery was performed. The patient was found to have recurrent lung metastasis and a pelvic pre sacral mass 18 months after surgery. He also had a chronic draining fistula in the right buttock which developed soon after surgery. A hard mass developed in the right buttock and on biopsy was found to be another site of metastasis. Treatment for metastatic disease had included FOLFOX /bevacizumab, lung radiation and isolated pelvic chemotherapy perfusion. A biopsy of the right buttock mass was performed with pathology consistent with the rectal primary (50% tumor cellularity).

Patient CLN4 is a 50 year old Hispanic male diagnosed with hemicolectomy for a T3N1 right sided colon cancer. The patient was found to have synchronous liver metastasis at the same time. He had undergone prior therapy with FOLFOX, FOLFIRI/bevacizumab and yittum-90 therapy to the liver. The patient entered the study 3 years after first diagnosis of metastatic disease and had tumors in the liver and a soft tissue mass in the epigastric area of the abdomen. A biopsy from the abdominal mass was consistent with the colon primary (30% tumor cellularity).

### Whole genome sequencing

For each patient tumor and germline DNA were sequenced to identify somatic alterations. WGS summary statistics are shown in Table 2. Aligned reads from both tumor and normal samples were evaluated for somatic events including non-synonymous single nucleotide variants (nsSNVs), indels, copy number variants (CNVs) and translocations. Circos plots in Additional files 1, 2 and 3 illustrate somatic events occurring in CLN2, CLN3 and CLN4 respectively [23].

**Table 2:** Whole genome sequencing

**Participant code**	**Total reads sequenced**	**Total bases sequenced**	**Coverage**	**Number of germline variants**	**Percent dbSNP**	**Transition/transversion ratio**
CLN2 Normal	1.9 billion	200 Gb	50X	2.3 million	88%	2.05
CLN2 Tumor	1.4 billion	146 Gb	42X	—		
CLN3 Normal	2.0 billion	210 Gb	62X	3.4 million	88%	2.06
CLN3 Tumor	1.96 billion	204 Gb	40X	—		
CLN4 Normal	0.97 billion	100 Gb	28X	3.3 million	88%	2.06
CLN4 Tumor	1.03 billion	107 Gb	31X	—		

The complete lists of somatic SNVs detected in the three evaluable specimens are provided in Additional file 4: SNVs and Indels. All three tumors contained KRAS mutations. Among the cancer genes mutated in patient CLN2 were *APC*, *KRAS*, *PIK3CA*, *SMAD4*, *MYST4*, *HUNK*, *INPPL1*, *TGFβ3*, and *TCF7L2*. Cancer genes mutated in CLN3 included *KRAS*, *INPP4B*, *PTPRE*, *CARD16* and *LRP2*. Cancer gene mutations in CLN4 were detected in *APC*, *KRAS*, *PIK3CA*, *KDR* and *AURKC* (Table 3). Some of the genes which were known to have non-synonymous mutations in colon cancer genes in prior studies were also seen in these samples. They include *APC*, *KCNQ5*, *KIAA1409*, *KRAS*, *LRP2*, *PIK3CA*, *SMAD4*, *TCF7L2* and *UHRF2*[9].

**Table 3:** SNVs and indels in relevance to cancer

**Sample**	**Chr**	**Gene name**	**Position**	**Coding event**	**Sequence change**	**Substitution**
CLN2	3	CPB1	150045158	SNV	G/A	R231Q
	6	ESR1	152423875	SNV	C/T	T431I
	10	TCF7L2	114907771	SNV	G/A	G424E
	11	INPPL1	71621016	SNV	A/G	E567G
	12	KRAS	25289548	SNV	C/T	G13D
	14	TGFβ3	75495381	SNV	G/T	Q381K
	18	BCL2	59136317	SNV	C/A	W188L
	18	PTPRM	8384547	SNV	G/A	V1415M
	10	MYST4	76460457	SNV	C/T	R1957W
	18	SMAD4	46858795	SNV	T/G	L540R
	19	SHANK1	55864301	SNV	G/A	R910C
	21	HUNK	32293208	SNV	G/A	R662Q
	3	PIK3CA	180399422	SNV	G/A	NA
	1	PTPRC	196985273	SNV	G/A	S852N
	13	MLNR	48693327	Indel	cccgg−/−ccgcc	Insertion
	2	SLC4A10	162427730	Indel	atcag−/−aaaa	Insertion
	6	UTRN	145111149	Indel	AAAT-g-GGAAA	Frameshift
	7	HNRNPA2B1	26202550	Indel	CAGAT-cctcc-TCTAA	Frameshift
CLN3	1	ARID1A	26973896	SNV	G/T	E1531
	2	LRP2	169845248	SNV	T/C	N400S
	3	MITF	70011193	SNV	C/G	S92C
	4	INPP4B	143222686	SNV	C/A	E864
	5	GPR98	89974441	SNV	G/T	V787L
	7	CYLN2	73409559	SNV	C/A	S344Y
	10	PTPRE	129758044	SNV	G/A	R369Q
	12	KRAS	25289551	SNV	C/T	G12D
	4	MAML3	26202550	Indel	AAAT-ctg-CTGCT	AA_Deletion
	6	PGC	140871034	Indel	CCTGC-aga-AGAGC	AA_Deletion
CLN4	1	ARID4B	233412380	SNV	C/A	R826M
	3	PIK3CA	180434779	SNV	A/G	H1048R
	4	KDR	55655861	SNV	G/A	R946C
	5	APC	112129944	SNV	G/T	G53V
	5	APC	112201150	SNV	C/T	Q654
	5	APC	112203580	SNV	G/T	E1464
	5	APC	112205360	SNV	A/G	K2057R
	6	PTCRA	42998820	SNV	G/T	V46F
	12	KRAS	25289552	SNV	C/A	G12C
	2	HOXD9	176696536	Indel	cagcc-/gcagc	Insertion

Non-synonymous SNVs found in the coding regions of genes were analyzed using the SIFT (Sorting Tolerant From Intolerant) algorithm to determine if such mutations may affect protein function [22]. Genes identified to have damaging effects on the protein product are indicated in Additional file 5: SIFT Predictions for SNVs and Indels. Of the 115 coding variants in CLN2, 61 were predicted to be damaging (53%); CLN3 had 90 coding variants of which 38 were damaging (42%); and CLN4 had 44 coding variants of which 20 were damaging (47%).

### Copy number analysis

Copy number analysis was performed using a sliding window comparison of coverage between tumor and normal using our in-house custom tool. Regions of gain or loss in the tumor samples are outputted as log2 ratios in Additional file 6: CNVs. CLN2 had whole chromosome copy number gain of chromosome 13, and chromosome 8q. Significant genes deleted in CLN2 include *TP53*, *BCL2*, *PIK3CA*, *SMAD2*, *SMAD3*, *SMAD4*, *APC2*, *DCC*, *TGFβ1*, *TCF3*, *TCF4* and *TCF12*. CLN3 exhibited whole chromosome copy number gain of chromosomes 1 – 5. A significant amplification occurred at 1pter and encompassed *NOCL2*, *PLEKHN1*, *SDF4*, *UBE2J2*, *CENTB5*, *CPSF3L*, *MXRA8*, *ATAD3B*, *ATAD3A*, *SSU7A*, *SLC35E2*, *NADK*, *GNB1*, *GABRD*, and *PRKCZ*. A focal amplicon at 12p contains *KRAS*, which is also mutated in this patient’s tumor. An interstitial deletion of about 16 Mb was seen in chromosome 13, which encompasses the *RB1* locus. Somatic copy number analysis in CLN4 was confounded by low tumor cellularity (30%); however we were able to detect several events including whole chromosome gain of chromosome 13.

Furthermore, several genes containing SNVs also mapped within regions of copy number change. A list of these genes has been included in Table 4. Notably in CLN2, *SMAD4* was deleted and harbored an L540R somatic mutation. Additionally, *PTPRM* was deleted and contained a V1415M nsSNV.

**Table 4:** Correlation of genes with SNVs and amplifications and deletions

**Sample**	**Chr**	**Gene name**	**SNV location**	**CNV variant type**	**Change associated with variant**	**Start**	**End**	**Correlation**
**CLN2**	8	ADAMDEC1	24312932	CNV-Loss	-1.098323504	24298000	24318700	-
	18	BCL2	59136317	CNV-Loss	-1.142019005	58946900	59136800	-
	17	C17orf39	17905985	CNV-Loss	-1.086327397	17886900	17909200	-
	13	DNAJC3	95127539	CNV-Gain	1.034261883	95159600	95201700	+
	18	DOK6	65495961	CNV-Loss	-1.080170349	65220300	65659400	-
	19	EEF2	3929160	CNV-Loss	-1.10433666	3927700	3935600	-
	13	MED4	47567121	CNV-gain	1.011225847	47549300	47562800	+
	15	MORF4L1	76965549	CNV-Loss	-1.25642835	76953100	76976400	-
	19	OR1M1	9065467	CNV-Loss	-1.185833041	9065000	9065800	-
	13	PCDH17	57105203	CNV-Gain	1.158413612	57105000	57140500	+
	8	PI15	75900191	CNV-Gain	1.077222314	75901700	75924000	+
	15	PML	72124335	CNV-Loss	-1.130706684	72074500	72122000	-
	18	PTPRM	8384547	CNV-Loss	-1.028666588	7637600	8387100	-
	19	PVRL2	50083243	CNV-Loss	-1.070323897	50042300	50073700	-
	19	SHANK1	55864301	CNV-Loss	-1.135111902	55859100	55907600	-
	18	SMAD4	46858795	CNV-Loss	-1.077097357	46827500	46855600	-
	15	THSD4	69844560	CNV-Loss	-1.12539172	69808500	69856700	-
	8	VPS13B	100723477	CNV-Gain	1.06690452	100098000	100957000	+
	19	XAB2	7594144	CNV-Loss	-1.039001256	7596500	7599800	-
	19	ZNF235	49483380	CNV-Loss	-1.210566986	49486200	49499300	-
	19	ZNF480	57510937	CNV-Loss	-1.12807401	57496100	57517900	-
	19	ZNF83	57808787	CNV-Loss	-1.330073623	57808100	57809600	-
**CLN3**	4	ANKRD17	74175875	CNV-Gain	1.050578956	74176700	74257700	+
	2	APOB	21086873	CNV-Gain	1.381146277	21078200	21119700	+
	1	ARID1A	26973896	CNV-Gain	1.088551435	26707600	26977900	+
	3	ATP13A5	194544518	CNV-Gain	1.074832063	194495900	194576400	+
	2	CLEC4F	70897420	CNV-Gain	1.474467603	70890000	70900800	+
	1	FNDC7	109063011	CNV-Gain	1.060116535	109071800	109085900	+
	3	GADL1	30871204	CNV-Gain	1.083837201	30750200	30822400	+
	4	GUCY1A3	156862650	CNV-Gain	1.051035883	156845700	156866700	+
	4	INPP4B	143222686	CNV-Gain	1.071823665	143169400	143560600	+
	2	IWS1	127960697	CNV-Gain	1.157172502	127955100	127999800	+
	3	KCNMB2	180028679	CNV-Gain	1.036705549	180021600	180027700	+
	12	KRAS	25289551	CNV-Gain	1.035184393	25274800	25275900	+
	2	LRP2	169845248	CNV-Gain	1.274350136	169693500	169926300	+
	3	LSG1	195872054	CNV-Gain	1.046959963	195844100	195855800	+
	1	MACF1	39573148	CNV-Gain	1.075279401	39322700	39722600	+
	3	MITF	70011193	CNV-Gain	1.055910933	70070200	70096500	+
	5	PAPD4	78951267	CNV-Gain	1.023426699	78969500	78969700	+
	2	PCBP1	70168678	CNV-Gain	1.147337373	70169200	70169400	+
	4	PDE5A	120666238	CNV-Gain	1.066043219	120655300	120742100	+
	1	PHTF1	114082912	CNV-Gain	1.075512922	114060600	114098600	+
	3	RPSA	39428561	CNV-Gain	1.099952073	39424200	39428600	+
	4	SH3BP2	2805228	CNV-Gain	1.208480128	2792500	2804000	+
	5	SLC6A19	1265596	CNV-Gain	1.142273516	1257300	1274700	+
	3	SMC4	161633513	CNV-Gain	1.02766824	161602500	161603100	+
	2	SMYD5	73305580	CNV-Gain	1.59092836	73301300	73306500	+
	1	SPOCD1	32038250	CNV-Gain	1.187067318	32029900	32040400	+
	1	ST6GALNAC3	76650550	CNV-Gain	1.08116566	76355200	76864700	+
	1	TMEM39B	32340671	CNV-Gain	1.071050645	32318800	32339600	+
	2	VPS54	63993297	CNV-Gain	1.158423232	63974100	64064600	+
	2	WDR35	20045755	CNV-Gain	1.180181947	19976800	20052800	+
	5	WWC1	167824543	CNV-Gain	1.083413977	167656200	167825900	+
CLN4	16	ABCC12	46688204	CNV-Gain	0.757287	46702800	46702800	+
	13	SPATA13	23761296	CNV-Gain	0.894588	23774800	23761296	+

### Impact of *INPPL1* silencing on cell proliferation in HCT116 cells

**Figure 1:**
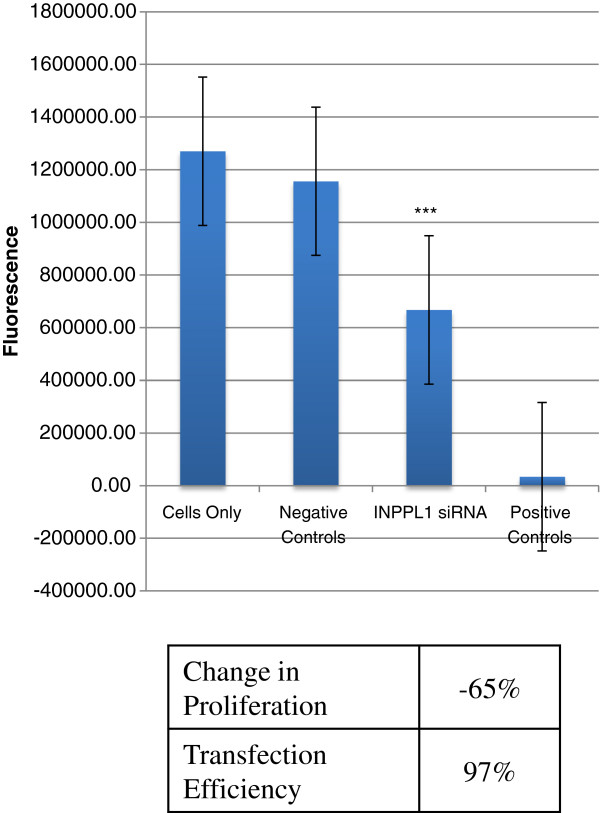
**Effect of INPPL1 siRNA on cell proliferation in HCT116 cells.** Changes in proliferation in HCT116 cells 72 hours post-transfection with pooled control or pooled INPPL1 sequences. Fluorescence data is shown for cells only (no siRNA sequences), negative controls (non-silencing siRNA sequences), INPPL1 siRNA, and positive controls (siRNA sequences targeting essential genes). The effects of INPPL1 siRNA are based on differences between average fluorescence signal generated by negative controls and INPPL1 siRNA sequences. Asterisks denotes statistical significance.

The PI3 kinase pathway has been previously associated with colorectal cancer, where *PIK3CA* mutations occur in approximately 15% of colorectal tumors [10,24]. Phosphatidyl-3,4,5-triphosphate (PI3,4,5P_3_), is a key phosphoinositide generated from PI3 kinase, which regulates PKB/AKT mediated cell survival and proliferation [25]. In our analysis, we identified a mutation in *INPPL1* (inositol polyphosphate phosphatase-like 1)*,* which encodes SHIP2, the phosphatase that plays an important role in the conversion of PI3,4,5P_3_ to PI3,4P_2._ The E567G mutation identified in the *INPPL1*gene was predicted to be damaging by SIFT, and validated by Sanger sequencing. Thus we investigated the impact of silencing *INPPL1* by RNA inhibition on cell proliferation. For this study, we used small interfering RNA (siRNA). HCT116 cells were seeded into 384-well plates containing siRNA buffer (no transfection), control siRNA sequences, or *INPPL1* siRNA sequences. At 72 hours post-transfection, cell proliferation was measured. Changes were measured based on differences between non-silencing control siRNA and *INPPL1* siRNA sequences (Figure 1). At 72 hours post-transfection, we detected a 65% decrease in HCT116 cell proliferation, suggesting that *INPPL1* may be required for CRC cell growth.

### Impact of *INPPL1* siRNA on protein expression of downstream signaling targets of PI3K -Western analysis

**Figure 2:**
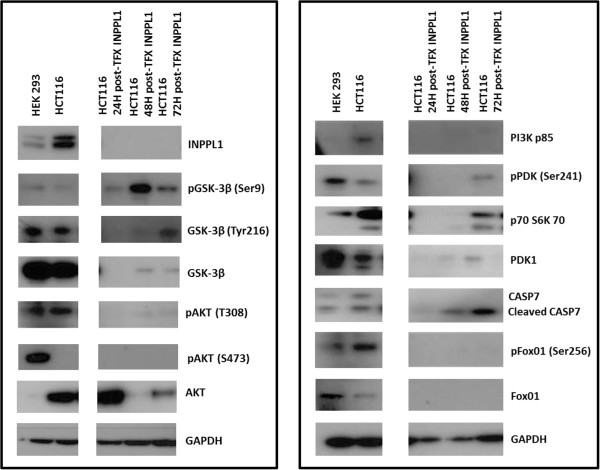
**Western blots.** Basal protein expression of HEK293 and HCT116 cells as well as changes in expression in HCT116 cells 24, 48, and 72 hours post-INPPL1 siRNA transfection. For post-transfection samples, cell lysates were treated with pooled INPPL1 siRNA sequences and harvested at each time point.

Since SHIP2 plays a role in PI3K/AKT signaling, *INPPL1* siRNA transfected and untransfected HCT116 cell lysates were used for Western blots and probed with antibodies to several downstream PI3K/AKT pathway targets as illustrated in Figure 2. HEK293 cell lysates were used as a positive control in our western blots, due to the ease of maintenance, abundance of proteins, and known expression of multiple proteins. As expected, a reduction in *INPPL1* protein levels was observed at 24, 48 and 72 hours post transfection. *INPPL1* siRNA treated HCT116 cells showed no expression of PI3K as early as 24 hours post transfection. Hence we looked at the protein levels of a series of downstream signaling targets of PI3K. For PDK1, both pPDK1 (Ser 241) and PDK1 antibodies were used. In the untransfected cell lines, pPDK and PDK1 were expressed. Similar to PI3K, *INPPL1* siRNA treated cells showed no expression of either the pPDK1 or PDK1 at 24 and 48 hours. AKT phosphorylation was detected at T308 and S473 in HEK293 cells, however, only phosphor-T308 was detected in HCT116 cells. Total AKT was detected at high levels in HCT116 cells. *INPPL1* siRNA transfected cells showed a marked reduction in the phosphorylation of AKT as seen in 24 and 48 hours post transfection. Moreover, *INPPL1* siRNA inhibited total AKT protein expression at 48 hours in HCT116 cells, with expression returning at 72 hours post transfection. *INPPL1* siRNA transfection also led to reduced p70 S6 kinase in the HCT116 cells within 24 hours post transfection. Phosphorylated FOX01 (Ser256) was detected in untransfected cells, but was significantly diminished in HCT116 cells transfected with *INPPL1* siRNA. Another downstream effector of AKT is GSK-3β, which upon phosphorylation by AKT at Ser9 becomes inactivated leading to increased cell cycle and β-catenin signaling. *INPPL1* siRNA led to decreased total GSK-3β at 24 hours in HCT116 parental cell lines. Interestingly, *INPPL1* knockdown in HCT116 cells led to increased phospho-(Ser9) GSK-3β in HCT116 cells at 48 hours. Finally, Caspase 7 activation, which is an indicator of apoptosis, also increased as indicated by the cleaved 35 kDa band in *INPPL1* siRNA transfected HCT116 cells. These results support inactivation of the PI3K/AKT pathway upon INPPL1 knockdown, suggesting a growth promoting role for *INPPL1* in colon cancer.

## Discussion

It is now established that the key mediators of the cancer cell phenotype are single base mutations, copy number alterations, translocations/rearrangements and epigenetic modifications of genomic DNA. Only now with the advent of NGS and bioinformatics capabilities can the entire human genome be interrogated for these changes. Importantly, with the development of targeted therapies, somatic genome analysis of tumors can shed light on possible therapeutically relevant events that might help inform treatment recommendations for advanced cancers.

In our small sample size, all three tumors had mutations in *KRAS. KRAS* mutations have been observed in 33% of the CRCs and are crucial for the early progression of adenoma to carcinoma in these tumors. Activating *KRAS* mutations in CRC tumor samples are an early event in the progression of colon carcinoma and the only predictive molecular marker useful for treatment decision as *EGFR* directed therapy is ineffective in the context of concomitant KRAS mutation [26]. However, even in the presence of *KRAS* wild type, therapy with either cetuximab or panitumumab is only effective in about 30% of cases suggesting that there are other molecular determinants of resistance. Additional genes harboring somatic mutations that have been characterized as cancer genes in our study include, *APC*, *TGFβ3*, *SMAD4*, *BCL2*, *INPPL1, INPP4B, PIK3CA*, *PTPRE* and *KDR*. Mutations in the *APC* gene have been identified in sporadic cancers and play a role in the *WNT*/β-catenin signaling pathway. Loss of *APC* function leads to β-catenin accumulation and binding to TCF/LEF transcription factors thereby activating *MYC* and *cyclin D1* leading to the transformation of the colon epithelium [27]. Thus *APC* mutations generally play a role in the initiation of colorectal cancers. *APC* also regulates cell proliferation of *RAS* induced *ERK* activation playing an important role in colorectal tumor suppression [28]. Colorectal tumors are known to have mismatch repair mutations that can increase the mutation rate in these tumors. However, one cannot also rule out the possibility that some of the mutations identified in these advanced cancers are not the result of previously administered chemo or radiation therapies.

Some notable CNVs were seen in these patients. CLN2 had amplification in the *MYC* oncogene. A number of known tumor suppressor genes were deleted in CLN2 such as *TP53*, *SMAD2*, *SMAD3*, *SMAD4*, *BCL2* and *TCF4*. SMAD4 alterations occur in over 50% of CRC and are believed to occur later in the course of disease [29]. *SMAD4* acts as a tumor suppressor to inhibit β-catenin [30] and targets the *TGFβ* signaling pathway to control epithelial cell growth [31]. Tumor CLN2 contained SMAD4 deletion and a concomitant SMAD4 mutation. Deletion of *BCL2* leads to an increase in the relapse of stage II colon cancers and could be a likely biomarker for therapeutic decisions [32]. Importantly, CLN2 also contained a somatic mutation in the *BCL2* gene. *TCF4* has been found to be mutated in variety of tumor types such as renal cell carcinoma, gastric carcinoma and breast cancer [33,34]. *TCF4* mutations have been reported in primary CRCs and its loss induces cell proliferation suggesting a possible role as a tumor suppressor [35-37]. CLN3 has a copy number gain encompassing the *VEGFA* locus, found in 3% of the cases and linked to higher tumor grade and vascular invasion and being an aggressive subgroup [38]. Copy number gain was also noted in *KDR* (*VEGFR-2*), a mediator of angiogenesis and its expression correlated with poor outcome in non-small cell lung carcinoma [39-42]. CLN4 had amplification of *EGFR*, *CDK8*, and *MIRH1. EGFR* mutations in the extracellular domain with gene amplifications are common in glioblastomas [43] and mutations in the tyrosine kinase domain with increased copy numbers are seen in lung carcinomas. However, amplification of *EGFR* seems to be an uncommon event in colorectal cancer [44,45]. *CDK8*, a cyclin dependent kinase amplified in *CLN4*, plays a major role in cell proliferation and PI3K inhibitors can be used in clinical trials for *CDK8*[46]. With regards to structural events in colon cancer, gene fusions of *TCF7L2* with *VTI1A* have previously been observed in 3% of the colon cancers [11]. We did not detect this translocation event in any of our tumors due to our sample size, however we did detected a novel *TCF7L2* (G424E) mutation in one of our tumors, suggesting that this gene can be perturbed by multiple mechanisms.

In our three CRC cases that underwent successful NGS, mutations were detected in genes and pathways that could possibly be therapeutically targetable. The *PI3K* pathway is recognized to be critical in cellular transformation, cell proliferation, adhesion, survival, and motility of cancer cells. In support of our observations, studies have shown that *PIK3CA* is mutated in up to 30% of CRC as well as other tumors such as breast, ovarian, and liver cancer [47] typically leading to activation of the PI3K/AKT/mTOR signaling pathway. Activated *PI3K* leads to an increase in the phosphoinositides PI3,4,5P_3_ and PI3,4P_2_ which in turn leads to phosphorylation of *AKT*. PI3,4,5P_3_ serves as a substrate for SHIP2 where it is converted to PI3,4P_2_. Both PI3,4,5P_3_ and PI3,4P_2_ have been shown to activate AKT phosphorylation [48]. However studies have shown that PI3,4P_2_ is more efficient than PI3,4,5P_3_ in binding to the PH domain of *AKT*, phosphorylating S473 and leading to membrane activation [25,49]. *In vitro* studies using phospholipid vesicles with PI3,4P_2_ alone were sufficient to activate *AKT*[50]. *INPPL1*, encodes SHIP2, the inositol phosphatase that converts PI3,4,5P_3_ to PI3,4P_2_. *SHIP2* is also a negative regulator of insulin signaling, plays an important role in EGF receptor turnover, and also has been reported to negatively regulate the *PI3K* pathway [51]. *INPPL1* has been shown to act as either a tumor suppressor or an oncogene in different tumor types. Furthermore, *SHIP2* expression has been associated with metastasis in breast cancer [52]. *INPPL1* mutations have been previously reported and occur in ~4% of colon tumors [53]. The *INPPL1* E567G mutation discovered in tumor CLN2 resides within the catalytic inositol polyphosphate 5-phosphatase domain that is critical to inositol phosphatase activity, and is predicted to be damaging by SIFT. Thus we performed additional functional genomic and mechanistic studies using RNAi to better understand the role of *INPPL1* in colon cancer cell growth and signaling. We used the HCT116 cell line model, which also harbors *PIK3CA* and *KRAS* mutations, similar to the patient containing the *INPPL1* mutation. As knockdown of *INPPL1* led to growth suppression, we hypothesize that this mutation may lead to a gain of function of INPPL1 thereby playing an oncogenic role in colon cancer as indicated by our *in vitro* studies. Upon *INPPL1* knockdown, we observed significant negative changes in phopho-signaling of key effectors of the *PI3K/AKT* pathway that suggest *INPPL1* might promote growth in colon cancer. This is an important insight as *SHIP2* converts PI3,4,5P_3_ to PI3,4P_2_, which has been shown to directly activate *AKT* independent of PI3,4,5P_3_. Studies by Fuhler *et al.* also show that treatment of multiple myeloma cells with *SHIP1/2* inhibitors causes cell arrest in the G2/M phase and induction of apoptosis via Caspase activation [54]. Therefore, additional studies of the role of SHIP2 in CRC are warranted, as this could provide alternative ways to approach inhibition of the *PI3K/AKT* axis as a means of treatment for a subset of colon cancer tumors.

## Conclusion

This study provides insights into the mutational landscape of metastatic recurrent colorectal cancer. *KRAS* being the most frequently mutated in human cancers with ~30% in colorectal cancers are the hallmarks in all these tumor samples. Several inhibitors for the downstream signaling targets of *RAS* such as *RAF* and *MEK* have not been very successful. *PI3K* inhibitors have been used in phase II clinical trials but have also not been very promising. There remains an urgent need to develop *KRAS* inhibitors to enhance treatment options in mCRC patients with *KRAS* mutations. Using an *in vitro* model with a colon cancer cell line, we have identified that an effective way of activating *AKT* signaling could be through PI3,4P_2_ and *INPPL1*. The inhibition of *INPPL1* may have a very significant role in cell proliferation and survival in colon cancer. And although specific recurrent mutations exist, our study further highlights therapeutically relevant contexts within metastatic colon tumors that might lead to new and improved ways to manage these difficult to treat tumors.

## Competing interests

The authors declare that they have no competing interests.

## Authors’ contributions

VS Analysis and interpretation of data, and drafting and revising the content of the manuscript. RR Principal Investigator, acquisition of patient samples, designing treatment options based on the sequencing results, drafting the clinical content in the manuscript. NL Cell proliferation studies and Western analysis. SS Creating the pipeline for the sequencing analysis and running the initial analysis. MC Acquisition of patient’s sample and study design. WL Library preparation and Illumina sequencing of the tumor and normal samples, guidance and training of additional staff for library preparation and sequencing. AK, TI, AC Sequencing analysis pipeline support. HB, LP Library preparation and Illumina sequencing of the tumor and normal samples. AB DNA isolation from the patient blood samples and tumor specimens. CM Validation of sequencing data. GH Determined tumor cellularity in the tumor samples and chipped the tumor sample for DNA isolation. DVH, DC Contributed to intellectual content of the paper. JC Principal Investigator and contributing to the intellectual content of the paper. All authors read and approved the final manuscript.

## Pre-publication history

The pre-publication history for this paper can be accessed here:

http://www.biomedcentral.com/1755-8794/7/36/prepub

## Supplementary Material

Additional file 1 **CLN2 Circos Plot.** Circos plot illustrating somatic events occurring in patient CLN2. Copy number changes are shown in the inner circle plot with red denoting copy number amplification and green denoting copy number deletion. Lines adjacent to gene names describe type of somatic event that a gene is involved in including somatic point mutation (blue), somatic indel (cyan). Click here for file

Additional file 2 **CLN3 Circos Plot.** Circos plot illustrating somatic events occurring in patient CLN3. Copy number changes are shown in the inner circle plot with red denoting copy number amplification and green denoting copy number deletion. Lines adjacent to gene names describe type of somatic event that a gene is involved in including somatic point mutation (blue), somatic indel (cyan). Click here for file

Additional file 3 **CLN4 Circos Plot.** Circos plot illustrating somatic events occurring in patient CLN4. Copy number changes are shown in the inner circle plot with red denoting copy number amplification and green denoting copy number deletion. Lines adjacent to gene names describe type of somatic event that a gene is involved in including somatic point mutation (blue), somatic indel (cyan). Click here for file

Additional file 4 SNVs and Indels. Click here for file

Additional file 5 SIFT Predictions for SNVs and Indels. Click here for file

Additional file 6 CNVs. Click here for file
