# Supplementary figures and images for: Whole genome sequencing reveals potential targets for therapy in patients with refractory KRAS mutated metastatic colorectal cancer
Source: BMC Med Genomics. 2014 Jun 18;7:36. doi: 10.1186/1755-8794-7-36 (PMC4074842; doi:10.1186/1755-8794-7-36)

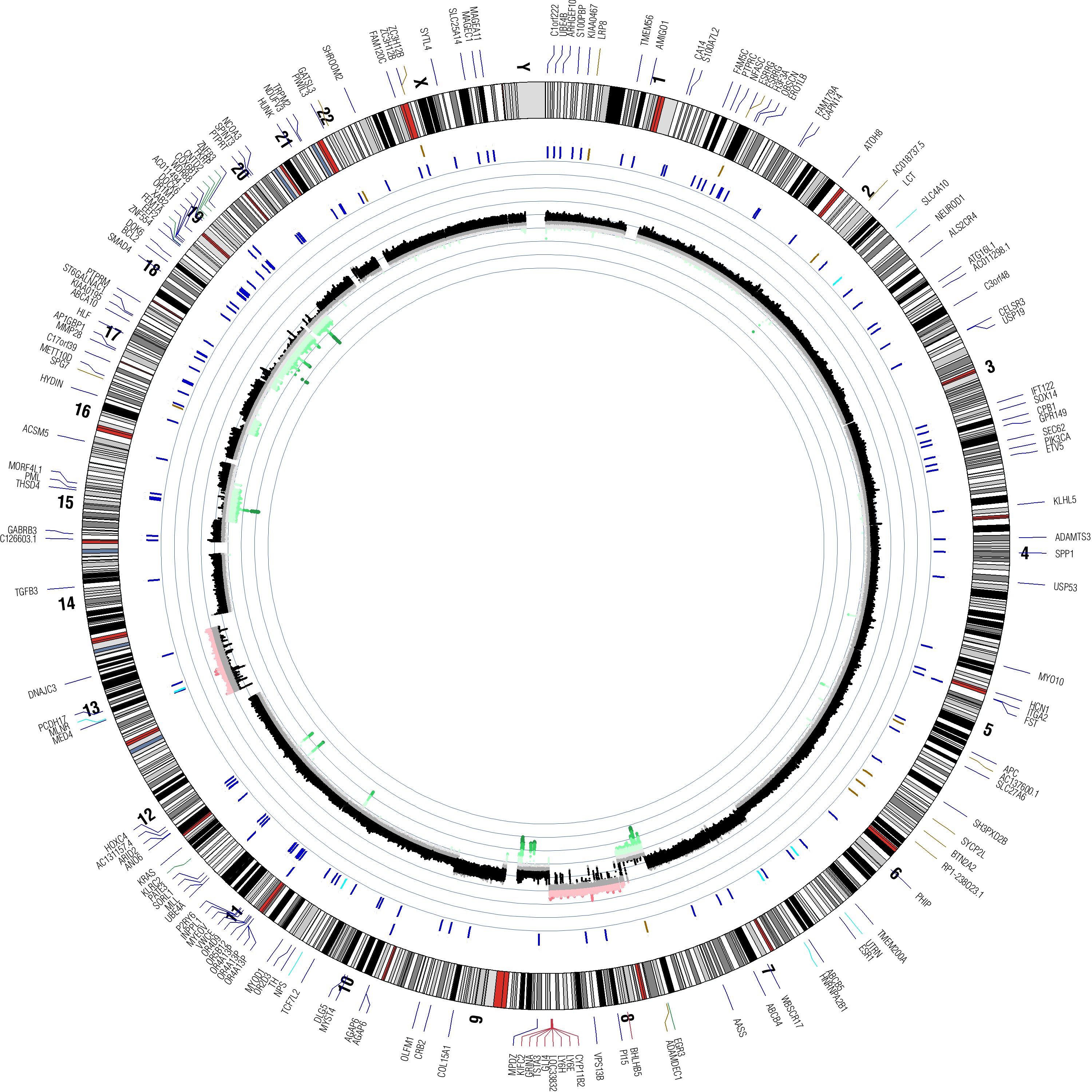

Supplement: Additional file 1 — CLN2 Circos Plot. Circos plot illustrating somatic events occurring in patient CLN2. Copy number changes are shown in the inner circle plot with red denoting copy number amplification and green denoting copy number deletion. Lines adjacent to gene names describe type of somatic event that a gene is involved in including somatic point mutation (blue), somatic indel (cyan). [file 1755-8794-7-36-S1.png]

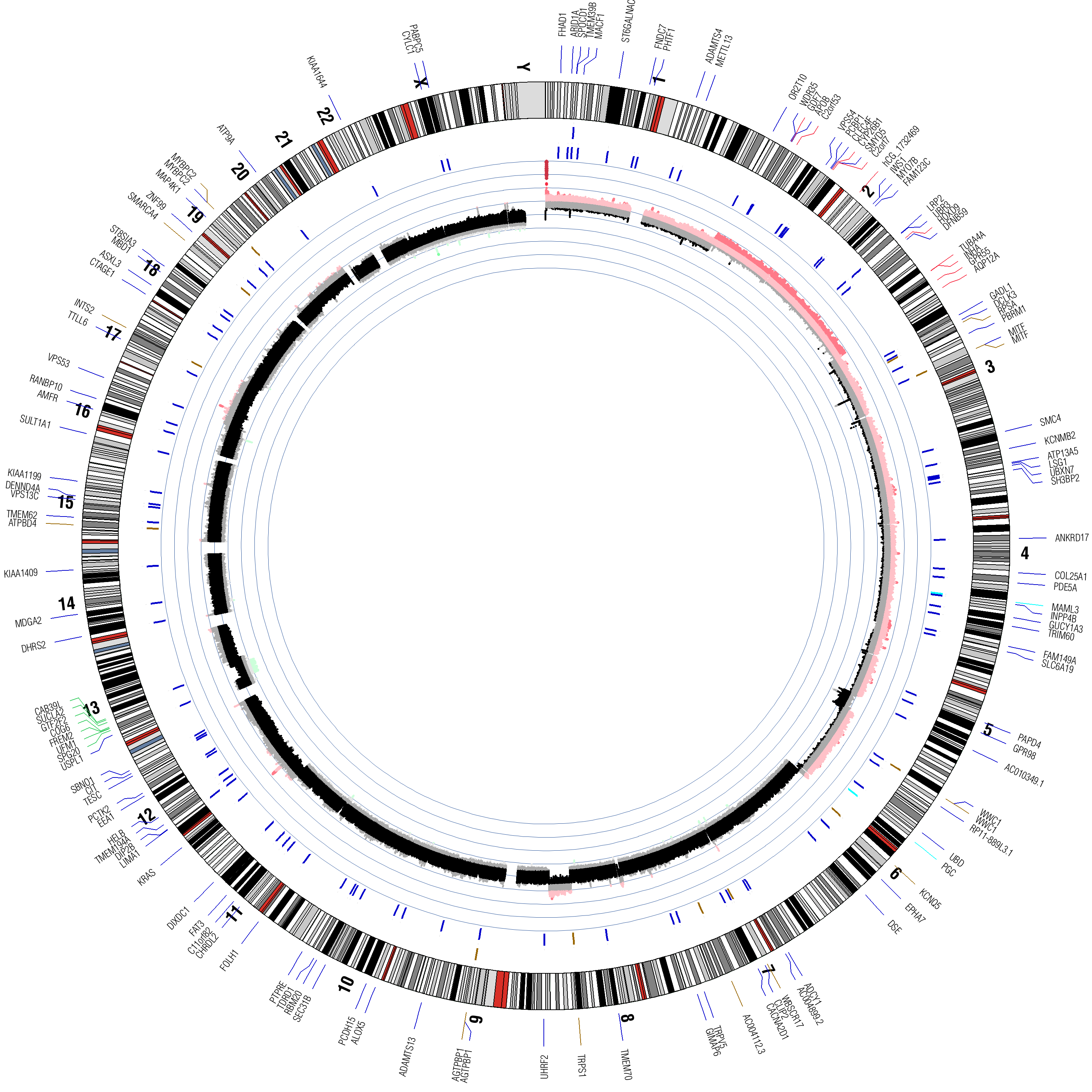

Supplement: Additional file 2 — CLN3 Circos Plot. Circos plot illustrating somatic events occurring in patient CLN3. Copy number changes are shown in the inner circle plot with red denoting copy number amplification and green denoting copy number deletion. Lines adjacent to gene names describe type of somatic event that a gene is involved in including somatic point mutation (blue), somatic indel (cyan). [file 1755-8794-7-36-S2.png]

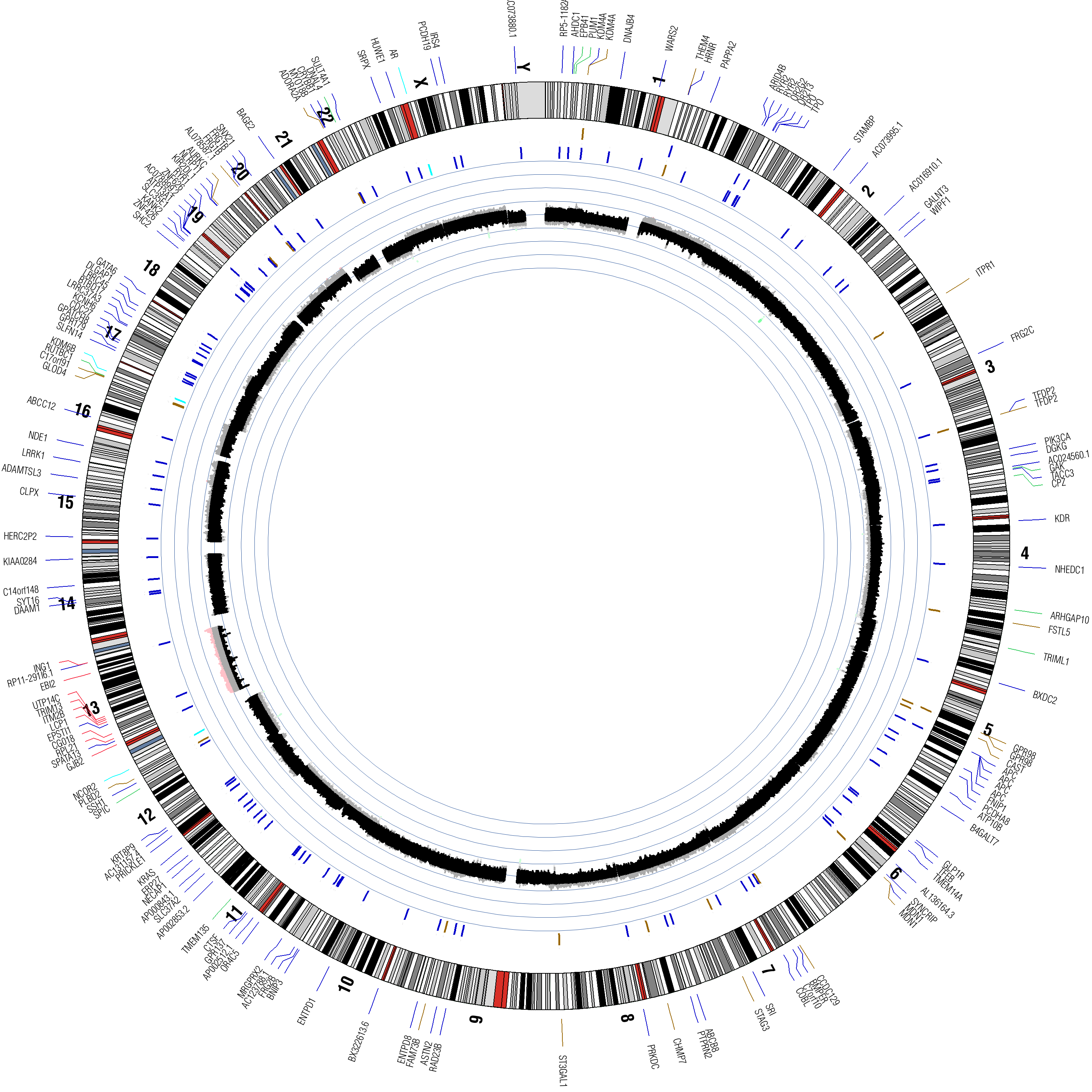

Supplement: Additional file 3 — CLN4 Circos Plot. Circos plot illustrating somatic events occurring in patient CLN4. Copy number changes are shown in the inner circle plot with red denoting copy number amplification and green denoting copy number deletion. Lines adjacent to gene names describe type of somatic event that a gene is involved in including somatic point mutation (blue), somatic indel (cyan). [file 1755-8794-7-36-S3.png]
